# d‐Electron Asymmetry‐Driven CN Coupling on Heteronuclear Dual‐Atom Catalysts for Sustainable Urea Electrosynthesis

**DOI:** 10.1002/advs.202511001

**Published:** 2025-07-29

**Authors:** Zaifu Jiang, Jingjing Wang, Dingmei Zhang, Panlong Kong, Xiaotao Zhang

**Affiliations:** ^1^ School of Mathematics and Physics Jingchu University of Technology Jingmen 448000 China; ^2^ Zhuhai Beijing Institute of Technology (BIT) Beijing Institute of Technology Zhuhai 519088 China

**Keywords:** C─N coupling, d‐electron asymmetry, dual‐atom catalysts, electrochemical urea synthesis, interpretable machine learning

## Abstract

The transition toward carbon‐neutral chemical manufacturing calls for innovative strategies to produce nitrogen‐based compounds with minimal environmental impact. Urea, a key nitrogen‐rich chemical, is currently synthesized via the energy‐intensive Bosch‐Meiser process, which relies heavily on fossil fuel‐derived ammonia. As a sustainable alternative, electrochemical urea synthesis (ECUS) enables the direct coupling of nitrogenous and carbonaceous precursors under ambient conditions, yet remains hampered by sluggish kinetics and poor selectivity—particularly in the critical C─N bond formation step. Here, density functional theory (DFT) calculations is integrated with data‐driven machine learning to systematically explore the activity landscape of nitrogen‐doped graphene‐supported dual‐metal‐atom catalysts (M′M@NC) for C─N coupling. A comprehensive reaction network is evaluated across 45 M′M@NC configurations, revealing three heteronuclear catalysts—VNi@NC, CoNi@NC and CoCu@NC—with consistently favorable thermodynamic and kinetic performance. Electronic structure analysis indicates that heteronuclear coordination promotes *CO activation and optimizes *NH_x_ adsorption, facilitating C─N coupling. Leveraging symbolic regression via the sure independence screening and sparsifying operator (SISSO) algorithm, interpretable descriptors linking C─N coupling energy to atomic‐level electronic properties is established, highlighting the critical role of d‐electron asymmetry. This results uncover fundamental design principles for dual‐atom catalysts and provide a predictive framework for guiding the development of next‐generation electrocatalysts for sustainable urea synthesis.

## Graphical abstract

Electrochemical urea synthesis is a promising route to sustainable nitrogen chemistry. This study combines quantum simulations and interpretable machine learning to uncover how dual‐metal atomic sites promote C‐N bond formation. Key catalysts are identified, and a simple electronic descriptor is built, offering design rules for next‐generation catalysts that couple CO_2_ and nitrogen species efficiently under ambient conditions.

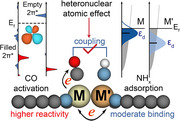

## 1 Introduction

The sustainable production of nitrogen‐based chemicals is a pressing global challenge, given their essential role in agriculture, pharmaceuticals, and materials science. Urea, a crucial nitrogen‐containing compound, is primarily synthesized via the Bosch‐Meiser process, which requires ammonia and carbon dioxide as feedstocks.^[^ ^1^, ^2^, ^3^, ^4^ ^]^ However, this conventional approach is highly energy‐intensive and heavily reliant on fossil fuel‐derived ammonia, contributing to significant carbon emissions. In the pursuit of carbon‐neutral chemical manufacturing, ECUS has emerged as an attractive alternative.^[^ ^5^, ^6^, ^7^, ^8^, ^9^, ^10^ ^]^ By directly coupling nitrogenous and carbonaceous precursors in aqueous media under mild conditions, ECUS has the potential to dramatically reduce energy consumption and mitigate environmental impacts. Despite its promise, the practical realization of ECUS remains hindered by sluggish reaction kinetics and poor selectivity, necessitating a deeper mechanistic understanding of the underlying catalytic processes.^[^ ^2^, ^11^, ^12^, ^13^ ^]^

Among the fundamental challenges of ECUS, the formation of the C─N bond represents a critical bottleneck that dictates both efficiency and selectivity.^[^ ^8^, ^10^, ^14^, ^15^, ^16^, ^17^ ^]^ The precise mechanism of C─N coupling remains an open question, with multiple competing pathways involving different nitrogenous intermediates, such as isocyanate (N─CO), carbamoyl (HN─CO), and carbamide (H_2_N─CO).^[^ ^5^, ^7^, ^18^, ^19^, ^20^, ^21^, ^22^ ^]^ Identifying the most reactive intermediate and the optimal reaction pathway remains elusive due to the dynamic nature of adsorption and activation processes on electrode surfaces. While single‐atom catalysts have demonstrated some capability for C─N coupling, they often suffer from inadequate binding energy modulation, leading to either excessive intermediate stabilization or undesired side reactions, such as hydrogen evolution and competitive nitrogen or carbon dioxide reduction.^[^ ^23^, ^24^, ^25^, ^26^, ^27^ ^]^ The rational design of advanced catalysts capable of promoting selective and efficient C─N bond formation is, therefore, imperative for advancing ECUS.

In this work, we combine DFT calculations with a data‐driven machine learning framework to systematically screen and evaluate nitrogen‐doped graphene‐supported dual‐metal‐atom catalysts for their ability to facilitate C─N coupling. A full reaction network comprising all plausible coupling steps was explored on 45 M′M@NC configurations, revealing three heteronuclear catalysts—VNi@NC, CoNi@NC, and CoCu@NC—with consistently favorable thermodynamic and kinetic performance across all coupling pathways. Detailed electronic structure analysis demonstrates that heteronuclear coordination enables synergistic effects, where one metal center enhances *CO activation while the other provides moderate *NH_x_ adsorption energy, thereby optimizing the C─N bond formation process. Furthermore, by applying the SISSO algorithm, we established interpretable mathematical descriptors that quantitatively correlate C─N coupling energy (E_coup_) with fundamental atomic features of the metal pairs. The resulting expressions highlight the critical role of d‐electron number variation between the two metal atoms in determining the coupling energetics. These findings not only deepen the mechanistic understanding of C─N coupling at dual‐atom interfaces but also provide a robust theoretical foundation for the rational design of next‐generation electrocatalysts for sustainable urea synthesis.

## 2 Results and Discussion

### 2.1 Screening Stable Electrocatalyst Models of M′M@NC

**Figure 1:**
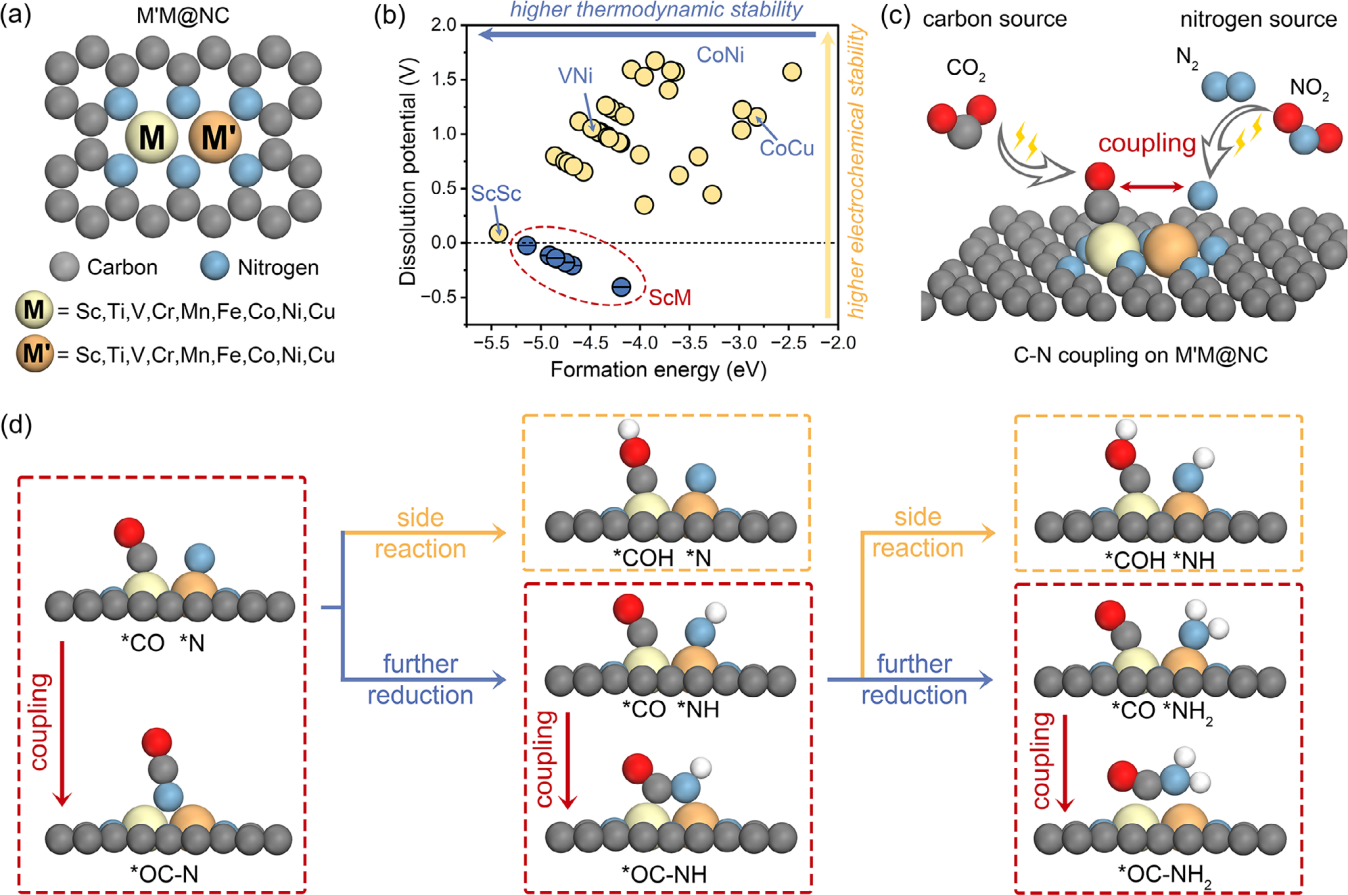
a) Geometric structures of the M′M@NC catalysts. b) The Formation energy (E_f_) and dissolution potential (U_diss_) of all considered M′M@NC systems. c) Schematic diagram showing the C─N coupling reaction on M′M@NC with different carbon and nitrogen source. d) Proposed C─N coupling pathway and associated side reactions during electrochemical urea synthesis. The red trajectories delineate the key C─N bond formation steps, while the yellow and blue pathways illustrate competing side reactions involving the hydrogenation of *CO to *COH and the progressive reduction of *N species to *NH_x_, respectively.

The modulated microenvironment of the active centers in dual‐atom catalysts (DACs) facilitates enhanced electronic interactions between the two atomic species, which is expected to improve their performance in C─N coupling reactions. **Figure** **1**a illustrates the schematic structure of nitrogen‐doped graphene‐supported dual‐metal‐atom catalysts, denoted as M′M@NC. In this study, we systematically selected metal dopants M and M′ from the fourth‐period transition metals, excluding zinc (Zn) due to its fully occupied 3d electronic configuration, resulting in a total of 36 heteronuclear and 9 homonuclear DACs. This selection enables a comprehensive investigation of their electronic properties and catalytic activity across diverse metal combinations. As shown in Figure 1a, the two transition‐metal atoms (M′M) in the DACs are embedded within a nitrogen‐doped graphene (NC) framework, where each metal atom is coordinated by three pyridinic sp^2^‐N atoms. Previous theoretical studies have identified this configuration as the most energetically favorable for stabilizing M′M dimers within N‐doped graphene.^[^ ^28^, ^29^, ^30^ ^]^ Furthermore, advanced characterization techniques have consistently confirmed the atomically dispersed nature of M′M species on the NC substrate and the presence of atomic pairs.^[^ ^30^, ^31^ ^]^ The large vacancy sites in the N‐doped graphene matrix provide sufficient anchoring space for transition‐metal atoms, while the unsaturated nitrogen atoms at the vacancy edges act as strong trapping sites for metal species. Simultaneously, the M′M unit exhibits high catalytic activity, attributed to charge redistribution, which plays a critical role in modulating electronic properties and facilitating electrocatalytic reactions.

We subsequently evaluated the thermodynamic and electrochemical stabilities of the 45 M′M@NC systems by calculating their formation energy (E_f_) and dissolution potential (U_diss_), as presented in Figure 1b and Table S2 (Supporting Information). Notably, most experimentally synthesized DACs were found to be both thermodynamically and electrochemically stable according to this evaluation criteria, demonstrating the reliability and feasibility of the indicators.^[^ ^32^, ^33^ ^]^ Encouragingly, the computed E_f_ values for all M′M@NC systems were negative, indicating the high thermodynamic stability on the NC substrate. Regarding electrochemical stability, eight systems (ScM@NC, excluding ScSc@NC) were excluded due to their negative U_diss_ values, indicating instability under electrochemical conditions. To further examine the feasibility of uniform metal dispersion, we calculated the binding energy (E_bind_ = E_M′M@NC_ – E_NC_ – E _M′_ – E_M_) and aggregation energy (E_agg_ = E_bind_ – E_coh_), where E_coh_ represents the cohesion energy of the metal atom in its bulk crystalline form.^[^ ^34^, ^35^ ^]^ The results, summarized in Table S3 (Supporting Information), demonstrate that all M′M dimers exhibit sufficiently negative E_bind_ and E_agg_, signifying the strong formation of M‐N bonds between the dispersed M′M dimers and the surrounding ligand environment. Based on these stability criteria, we ultimately identified 37 M′M@NC systems as promising candidates for further investigation.

### 2.2 C─N Coupling Pathways and Activities

Electrochemical urea synthesis typically involves the co‐reduction of carbon‐ and nitrogen‐containing reactants such as CO_2_ or CO (carbon sources), and NO_2_^−^ or N_2_ (nitrogen sources) (Figure 1c). To evaluate the initial adsorption behavior of these reactants, we computed the adsorption energies (E_ads_) for CO_2_, CO, NO_2_^−^, and N_2_ on M′M@NC catalysts. In general, most catalysts exhibited strong adsorption affinity toward both C‐ and N‐containing species, suggesting effective capture and activation of the reactants (Figures S1–S4, Supporting Information). However, MnNi@NC exhibited a positive adsorption energy for CO_2_, indicating weak or unfavorable binding. Similarly, NiNi@NC, NiCu@NC, and CuCu@NC showed positive adsorption energies for N_2_, implying limited potential for nitrogen activation in these systems. The activity of the C─N coupling step was assessed by computing the E_coup_ associated with the coupling process. A lower E_coup_ indicates a more thermodynamically favorable coupling event. Based on prior experimental observations and theoretical insights, *CO was selected as the sole representative of carbon‐containing intermediates, as it is the most common and stable electroreduction product from CO_2_ or CO under reaction conditions.^[^ ^14^, ^15^ ^]^ For nitrogen‐containing species, we considered *N, NH, and NH_2_ intermediates, which have been identified in previous studies as the most thermodynamically stable and kinetically accessible species formed during the electrochemical reduction of N_2_ or NO_2_^−^.^[^ ^16^, ^17^ ^]^ In contrast, DFT calculations reveal that OC─NO_x_ intermediates are intrinsically unstable on M′M@NC‐type surfaces and spontaneously dissociate, indicating that such pathways are unlikely to contribute to effective C─N bond formation under the studied conditions. An additional major challenge in achieving selective C─N bond formation lies in suppressing competitive side reactions. In particular, *CO can undergo further electrochemical reduction, forming species such as COH, which diverts the reaction pathway away from urea synthesis and toward undesired byproducts. Therefore, considering all the factors mentioned above, in this work we conduct a comprehensive theoretical investigation of the C─N coupling process on M′M@NC catalysts, aiming to identify favorable coupling pathways and suppress competing reactions. As illustrated in Figure 1d, we systematically mapped all considered C─N coupling pathways, focusing on the thermodynamic and kinetic accessibility of each potential coupling step between *CO and the relevant N‐containing intermediates.

**Figure 2:**
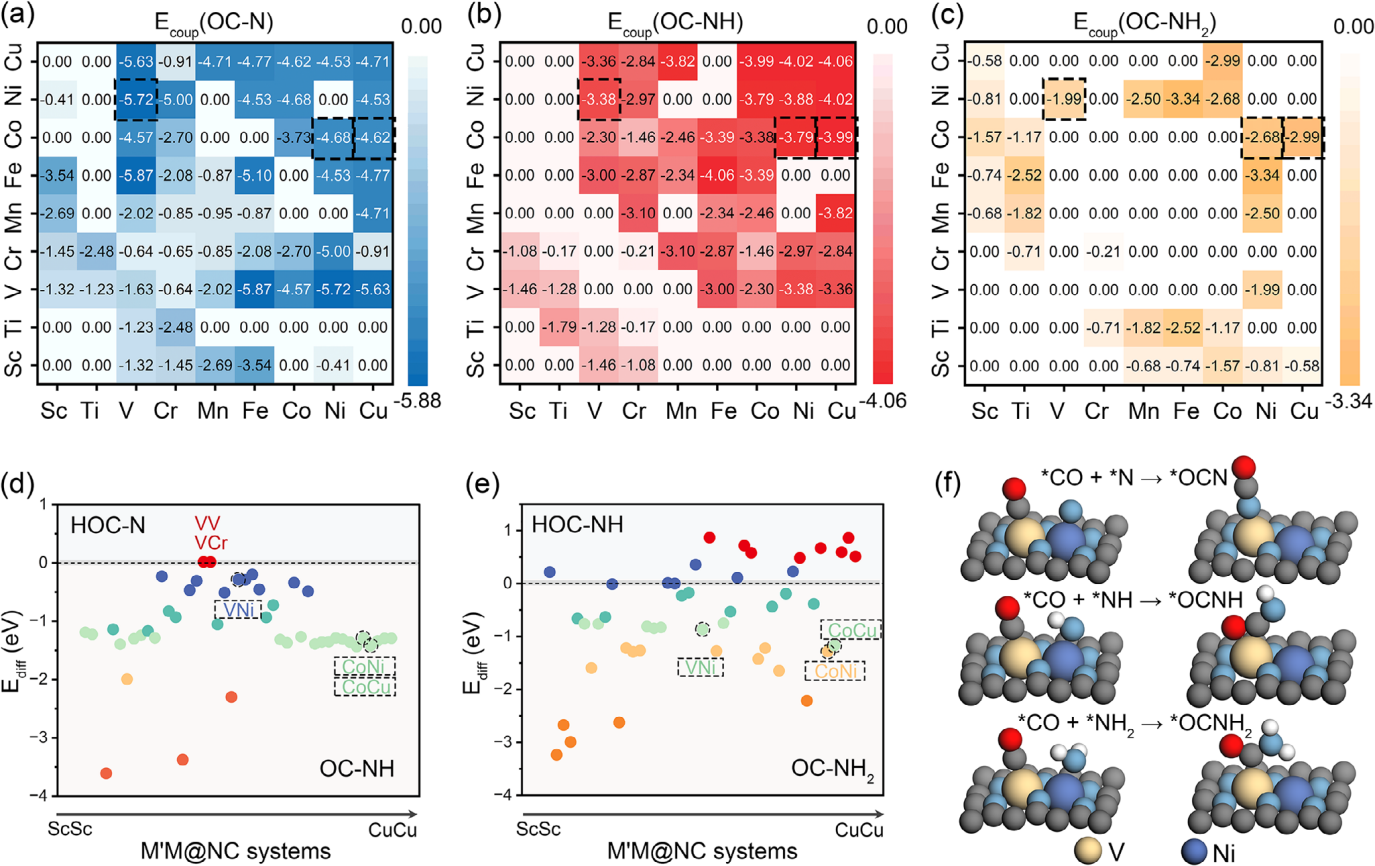
C─N E_coup_ of *CO with a) *N b) *NH and c) *NH_2_. Energy difference (E_diff_) between C─N coupling product and side product for d) OC─NH and e) OC─NH_2_. f) Optimized coupling configurations of *CO and *NH_x_ on VNi@NC catalysts.

The E_coup_ for all considered reaction steps were calculated through rigorous structural optimization, incorporating all available adsorption configurations on each catalyst surface (**Figure** **2**a–c). For certain systems, however, the coupling reactions were found to be thermodynamically or geometrically unstable, or the target products could not be formed. In these cases, the corresponding E_coup_ values were set to zero, indicating the inaccessibility of a viable coupling pathway under the given constraints. Among the 45 M′M@NC systems evaluated, 31 catalysts exhibited feasible OC─N coupling, while 26 and 14 catalysts enabled OC─NH and OC─NH_2_ coupling, respectively. The decreasing number of accessible pathways from *N to *NH_2_ can be attributed to the greater structural flexibility of hydrogenated nitrogen intermediates, which increases the complexity of the reaction network and introduces steric constraints on favorable coupling geometries.

To further understand the selectivity between target C─N coupling and competing side reactions, we computed the energy difference between the final products of these two processes, defined as E_diff_ = E_pro_coup_ – E_pro_side_, where E_pro_coup_ denotes the total energy of the target C─N coupling product, and E_pro_side_ corresponds to the total energy of the competing side product. A negative E_diff_ value indicates thermodynamic preference for C─N bond formation over undesired reaction pathways. As shown in Figure 2d,e, for the OC─NH coupling pathway, the majority of M′M@NC catalysts exhibit negative E_diff_ values, indicating favorable selectivity toward the desired coupling product. Notable exceptions include VV@NC and VCr@NC, which tend to favor the competing side reaction. In contrast, for OC─NH_2_ coupling, 14 catalysts demonstrate positive E_diff_, suggesting that selectivity decreases as the degree of hydrogenation increases. This trend underscores the inherent challenge of achieving selective C─N bond formation with highly hydrogenated nitrogen intermediates. Interestingly, we observe that M′M@NC catalysts composed of later transition metals generally exhibit stronger coupling abilities. This trend appears to correlate with their electronic density and d‐band electronic characteristics, indicating that electronic structure effects play a critical role in tuning C─N coupling reactivity and merit further investigation.

**Figure 3:**
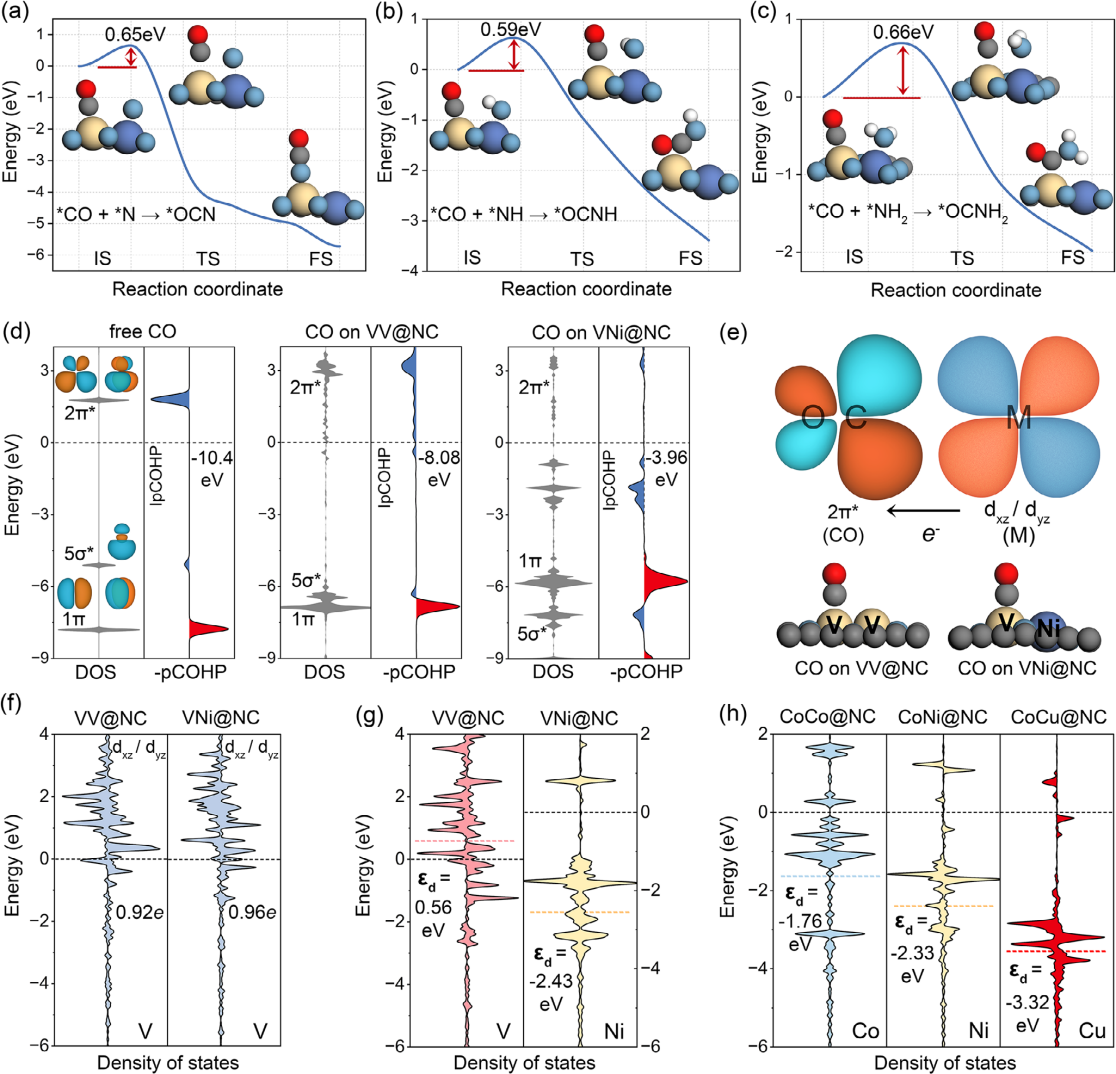
Free‐energy diagram for a) OC─N b) OC─NH and c) OC─NH_2_ coupling process on VNi@NC with climbing‐image nudged elastic band approach (CI‐NEB). Transition‐state (TS) energy barriers are indicated by arrows. d) Electronic density of states (DOS) and projected crystal orbital Hamilton population (pCOHP) of CO in free, on VNi@NC and on VNi@NC with integrated pCOHP (IpCOHP) values provided. e) Schematic illustration of π‐backdonation from the metal d_xz/yz_ orbitals to the antibonding 2π orbital of CO. f) Projected DOS (PDOS) of V atom in VV@NC and VNi@NC and the number of electrons occupied in d_xz/yz_ orbitals. g) DOS of V atom in VV@NC and Ni atom in VNi@NC with corresponding ε_d_ values. h) DOS of Co atom in CoCo@NC, Ni atom in CoNi@NC and Cu atom in CoCu@NC with corresponding εdε_d_ values.

Based on a comprehensive evaluation of both reaction activity and product selectivity, three catalysts (VNi@NC, CoNi@NC, and CoCu@NC) exhibited consistently favorable C─N coupling performance across all considered reaction pathways (Figure 2a–c,f; Figures S5 and S6, Supporting Information). To evaluate the thermal stability of the selected catalysts, ab initio molecular dynamics simulations were performed at 300 K (Figure S7, Supporting Information). All systems retained their initial structural configurations with only minor distortions observed over the course of the simulation, indicating good thermal stability under ambient conditions. Beyond evaluating the thermodynamic feasibility of the C─N coupling reactions, we further investigated their kinetic profiles (**Figure** **3**a–c; Figure S8, Supporting Information). The calculated activation energy barriers for all coupling steps were found to lie within the range of 0.29–0.86 eV. These values are comparable to—or even lower than—those reported for experimentally realized systems,^[^ ^7^, ^36^, ^37^ ^]^ highlighting the kinetic accessibility of the coupling processes. This result strongly supports the catalytic efficacy of the dual‐atom catalysts identified through our screening. Given the complexity of urea synthesis, in which reaction mechanisms and potential‐determining steps can vary significantly depending on the specific pathway and catalyst, the identification of catalysts capable of facilitating all possible C─N coupling steps is a notable achievement, highlighting their versatility and broad applicability. Notably, all three candidates are heteronuclear DACs, underscoring the importance of atomic‐level asymmetry in modulating catalytic behavior. In contrast, their corresponding homonuclear analogues, such as VV@NC, CoCo@NC, and NiNi@NC, demonstrate limited coupling ability, failing to support the full range of desired C─N coupling steps. This striking difference highlights the potential role of electronic asymmetry and synergistic effects in enhancing catalytic performance. Accordingly, we proceeded to investigate the underlying electronic structure of these systems to better understand the origin of their superior activity and selectivity.

### 2.3 Activity Origin of M′M@NC Toward C─N Coupling From the Electronic Structure

To uncover the electronic origins of C─N coupling activity, we focused our analysis on a subset of representative dual‐atom catalysts. NiNi@NC and CuCu@NC were excluded from further discussion due to their inability to adsorb nitrogen molecules, as previously established. We therefore concentrated on comparing homonuclear versus heteronuclear configurations, specifically: VV@NC versus VNi@NC, and CoCo@NC versus CoNi@NC and CoCu@NC. We first examine the VV@NC and VNi@NC pair. Analysis of their C─N coupling energetics reveals that *NH and *NH_2_ species are unable to effectively couple with CO on VV@NC. Since the adsorption of *NH_x_ intermediates and their coupling with *CO occur on competing sites, the underlying mechanism is governed by the relative binding affinities and surface reactivity of each species. We therefore began by evaluating the reactivity of adsorbed *CO on both catalysts. Our calculations show that CO on VNi@NC is significantly more activated than on VV@NC (Figure 3d). This is attributed to stronger π‐backdonation from the metal center to the antibonding 2π orbital of CO, which weakens the internal C─O bond and promotes reactivity (Figure 3e). This effect is quantitatively supported by the IpCOHP values of the C─O bond, which increase from −10.4 eV for free CO, to −8.04 eV on VV@NC, and further to −3.96 eV on VNi@NC (Figure 3d). These results highlight the critical role of the heteronuclear Ni atom in enhancing CO activation. To understand the electronic origin of this effect, we further examined the orbital occupancy of the V atom in the two configurations. When paired with a Ni atom, the V center in VNi@NC exhibits increased occupation of the d_xz/yz_ orbitals (Figure 3f), facilitating greater electron donation into the CO 2π* orbital. This enhances CO activation, making it more reactive for subsequent coupling steps. Meanwhile, we evaluated the binding characteristics of NH_x_ intermediates on the second metal site. Calculations of the d‐band center (ε_d_) reveal that the Ni atom, a later transition metal, exhibits a lower ε_d_ (−2.43 eV) compared to the V atom (0.56 eV) (Figure 3g). According to the d‐band center theory, this implies that *NH_x_ binds weaklier to Ni than to V, making it easier for *NH_x_ to desorb from Ni and migrate toward the activated *CO on the adjacent V site. This asymmetry in adsorption strength facilitates the spatial proximity and reactivity necessary for effective C─N coupling.

This mechanistic picture is further supported by examining the Co‐based systems, specifically CoCo@NC, CoNi@NC, and CoCu@NC. In these systems, the Co atom's d_xz/yz_ orbitals are fully occupied, resulting in similar CO activation capacity across all three catalysts. Hence, the key determinant of C─N coupling activity shifts to the adsorption strength of NH_x_ on the secondary metal site. As shown in Figure 3h, the d‐band centers of Co, Ni, and Cu are calculated to be −1.76, −2.33, and −3.32 eV, respectively. This trend aligns with our expectations: *NH_x_ species bind more weakly to Co and Cu, making them more accessible for coupling with *CO, and thus enabling enhanced C─N coupling activity in CoNi@NC and CoCu@NC relative to CoCo@NC. Notably, the CoCu@NC catalyst was successfully synthesized and demonstrated outstanding electrochemical performance, achieving a urea yield of 1.04 mol h^−1^ g_CuCo_^−1^ at a low applied potential of −0.45 V versus the reversible hydrogen electrode (RHE).^[^ ^38^ ^]^ This further supports the reliability of our findings. This experimental realization substantiates the robustness and practical relevance of our theoretical predictions. Collectively, these findings offer a comprehensive mechanistic understanding of how heteronuclear coordination environments modulate C─N coupling efficiency, highlighting their critical role in the rational design of high‐performance urea electrosynthesis catalysts. We find that combining early and late transition metals yields a synergistic electronic structure that promotes coupling bond formation. Specifically, the early transition metal site is more effective in activating the CO molecule through π‐backdonation, while the late transition metal offers moderate NH_x_ adsorption strength, in line with the Sabatier principle. This balance enables *NH_x_ to efficiently couple with activated *CO, thereby completing the C─N bond formation. Overall, this highlights the rational electronic design strategy enabled by heteronuclear dual‐atom catalysts for advancing electrochemical urea synthesis.

### 2.4 Identification of a Data‐Driven Descriptor for C─N E_coup_

**Figure 4:**
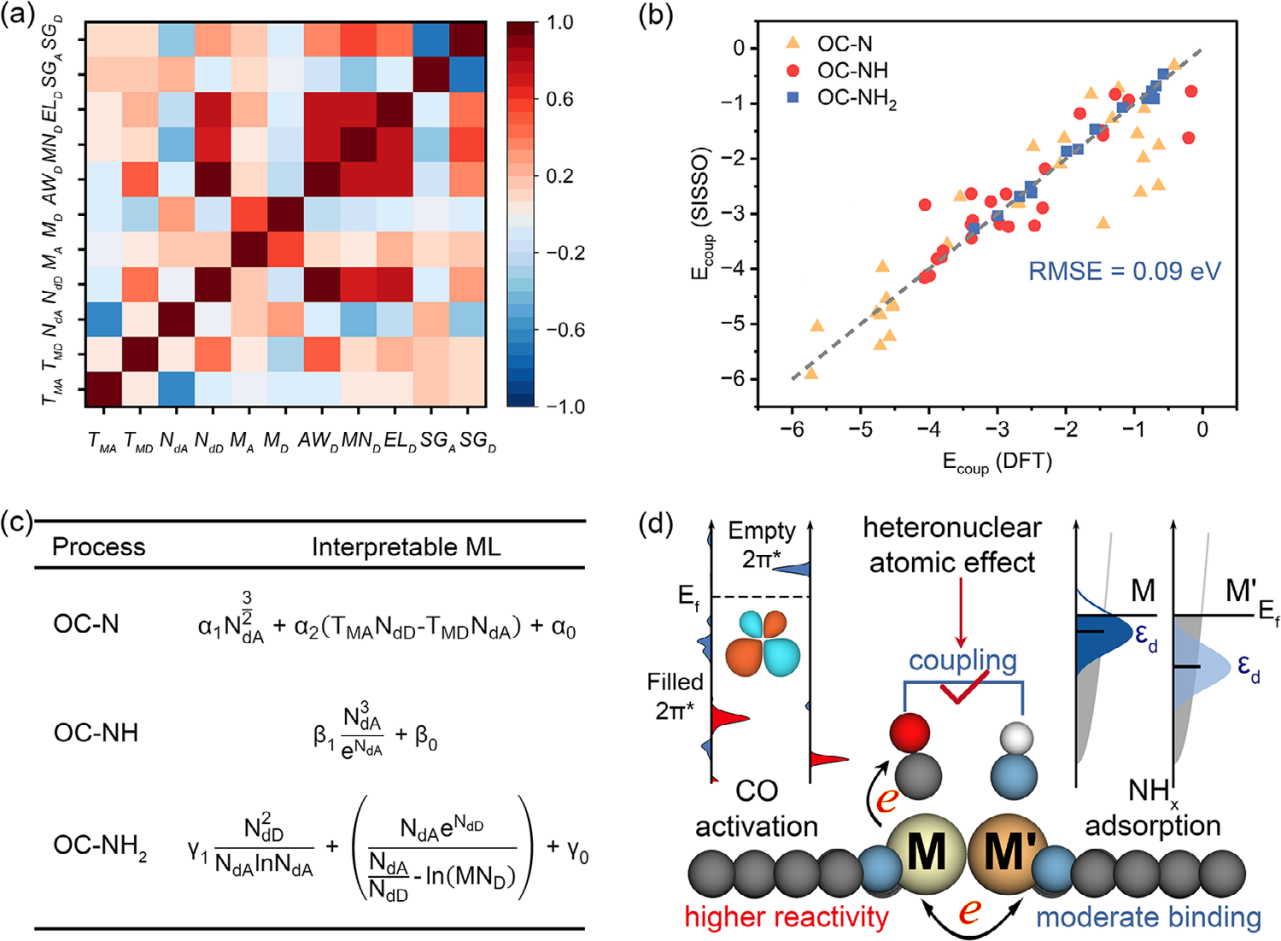
a) Pearson correlation matrix of the selected 11 features, where the subscript “A” denotes feature‐averaged values, and “D” indicates deviation values obtained by mean subtraction. These features include Melting Temperature (T_MA_, T_MD_), d valence electron (N_dA_, N_dD_), Magmom (M_A_, M_D_), Atomic Weight (AW_D_), Mendeleev Number (MN_D_), Pauling Electronegativity (EL_D_) and SpaceGroup Number (SG_A_, SG_D_). b) Comparison between the SISSO‐predicted and DFT‐calculated E_coup_ values in the training set. c) Derivable formulas identified through interpretable machine learning SISSO program for E_coup_ in all considered C─N coupling processes. d) Schematic illustration of the role of heteronuclear coordination in enhancing C─N coupling efficiency.

To quantitatively establish a predictive relationship between the C─N E_coup_ and the intrinsic physicochemical properties of M′M@NC catalysts, we employed a data‐driven machine learning framework. Specifically, we utilized the SISSO algorithm to identify a physically interpretable functional form of E_coup_. To construct a physically meaningful model, we performed symbolic regression over a comprehensive set of 48 primary descriptors, representing metal‐related properties known to govern catalytic behavior (Table S4, Supporting Information).^[^ ^15^, ^39^ ^]^ In particular, we emphasized the synergistic interactions between the two metal centers in heteronuclear dual‐atom systems. To encode these cooperative effects, we pre‐processed the primary features by applying arithmetic operations—averaging and subtraction—across the two metal sites, thereby capturing the bimetallic character of each catalyst. Through backward feature elimination and cross‐validation, we retained 11 highly independent and physically relevant features. Their pairwise correlations, quantified via Pearson similarity coefficients, are illustrated in **Figure** **4**a. We then conducted an exhaustive search through more than 300 million candidate expressions composed of these refined features using simple mathematical operations to ensure interpretability and generalizability of the resulting model. An optimal 2D descriptor was ultimately identified using compressed sensing techniques (Table S6, Supporting Information).

The resulting model demonstrates excellent predictive performance, achieving a root mean square error (RMSE) as low as 0.09 eV (Figure 4b), which is well within the accepted margin of uncertainty in first‐principles calculations. Notably, both components of the final descriptor are intrinsically linked to d‐electron characteristics: the average number of d‐valence electrons (N_dA_) and the deviation in d‐valence electrons between the two metal centers ((N_dD_). These features enter the model through rapidly varying functional forms—such as cubic and exponential terms—highlighting their critical influence in governing E_coup_ (Figure 4c). The explicit analytical expression derived from interpretable machine learning not only reinforces the pivotal role of d‐electron configuration disparities in dictating the energetics of C─N coupling but also exhibits strong consistency with our prior mechanistic insights. The generality of this principle is further substantiated by a range of reported heterostructure catalysts—such as Ta_2_W_2_C_3_ MXenes,^[^ ^7^ ^]^ Pd_1_Cu_1_‐TiO_2_,^[^ ^40^ ^]^ and Mo‐PCN‐222(Co) tandem catalyst^[^ ^41^ ^]^—all of which exhibit notable activity in electrochemical urea synthesis, underscoring the essential function of d‐electron modulation in governing catalytic performance. As illustrated in Figure 4d, heteronuclear coordination enables the tuning of local electronic environments to achieve both enhanced CO activation and moderate NH_x_ adsorption, thereby accelerating C─N bond formation and promoting overall urea synthesis. Given the inherent complexity of the C─N coupling mechanism, the strong agreement between our data‐driven model and existing theoretical and experimental insights underscores the utility of interpretable machine learning for rational catalyst design. This framework offers a transferable and physically grounded approach to guide the engineering of C─N coupling for next‐generation electrocatalysts in sustainable nitrogen chemistry.

## 3 Conclusion 

This work addresses a central challenge in electrochemical urea synthesis—efficient and selective C─N bond formation—by combining first‐principles calculations and data‐driven modeling to uncover the activity origins of dual‐atom catalysts. Through a systematic evaluation of 45 M′M@NC surfaces, we identify three heteronuclear catalysts—VNi@NC, CoNi@NC, and CoCu@NC—that exhibit promising thermodynamic and kinetic performance across all relevant coupling pathways. Detailed electronic structure analysis reveals that heteronuclear coordination fosters a favorable balance: enhanced *CO activation by early transition metals and moderate *NH_x_ binding by later metals. This synergy lowers reaction barriers and improves selectivity toward C─N bond formation. Meanwhile, our use of the SISSO machine learning framework enables the identification of physically interpretable descriptors that link catalytic performance to fundamental electronic structure, with d‐electron number variation emerging as a key factor. These insights not only deepen the mechanistic understanding of C─N coupling on complex catalytic interfaces but also provide a rational design strategy for tuning metal‐support interactions. By illuminating the electronic principles that govern reactivity in dual‐atom catalysts, this study paves the way toward scalable, efficient, and environmentally benign routes for sustainable nitrogen chemistry.

## 4 Computational Details

DFT calculations are performed using the Vienna ab‐initio simulation package (VASP) with the projector augmented‐wave method.^[^ ^42^, ^43^ ^]^ The exchange correlation is approximated with Perdew‐Burke‐Ernzerhof functional.^[^ ^44^ ^]^ The electronic self‐consistency energy and force convergence criterion are chosen as 10^−6^ eV and ‐10^−3^ eV/Å, respectively. The supported substrate is a graphene supercell with lattice a, b equal 12.4 and 12.7 Å where four carbon atoms are removed and six carbon atoms around the vacancy are replaced with nitrogen. A Monkhrost‐Pack scheme with a 3  ×  3  ×  1 k‐point grid is used to sample the Brillouin zone, and a plane wave cutoff of 520 eV is used for all calculations. A vacuum layer of 20 Å was used to eliminate the interactions between periodic images. VASPsol code was used to simulate the aqueous environment with the relative permittivity of the electrolyte set to 78.4.^[^ ^45^, ^46^ ^]^ To account for the electron correlation of 3d orbitals, a Hubbard correction of U is employed (Table S1, Supporting Information).^[^ ^47^, ^48^, ^49^ ^]^ Van der Waals interactions were described with the zero damping DFT‐D3 correction scheme.^[^ ^50^ ^]^

In this study, we employed linear regression analysis in combination with the SISSO algorithm to derive physically interpretable expressions for the target output.^[^ ^51^, ^52^, ^53^ ^]^ SISSO selects optimal descriptors from a high‐dimensional feature space by balancing model simplicity and predictive performance.^[^ ^15^, ^54^, ^55^, ^56^ ^]^ To prevent overfitting and ensure robustness, we applied tenfold cross‐validation. In this procedure, the dataset is partitioned into ten subsets, with nine used for training and one for validation in each iteration. The average RMSE across all folds was used to evaluate model accuracy and generalizability. Additionally, a Pearson correlation matrix was constructed to assess the degree of linear dependence among features, facilitating the identification of mutually independent variables for inclusion in the final model. More details about the computational method could be found in the Supporting Information.

## Conflict of Interest

The authors declare no conflict of interest.

## Supporting information



Supporting Information

## Data Availability

The data that support the findings of this study are available from the corresponding author upon reasonable request.
