# Supplementary material for: d‐Electron Asymmetry‐Driven CN Coupling on Heteronuclear Dual‐Atom Catalysts for Sustainable Urea Electrosynthesis
Source: Adv Sci (Weinh). 2025 Jul 29;12(40):e11001. doi: 10.1002/advs.202511001 (PMC12561354; doi:10.1002/advs.202511001)
Supplement: Supplementary file 1 — Supporting Information [file ADVS-12-e11001-s001.pdf]

Supporting Information

d-Electron Asymmetry-Driven C-N Coupling on Heteronuclear Dual-Atom Catalysts for Sustainable Urea Electrosynthesis

Zaifu Jiang[1,*], Jingjing Wang[1], Dingmei Zhang[1], Panlong Kong[1], Xiaotao Zhang[2]

[1] *School of Mathematics and Physics, Jingchu University of Technology, Jingmen 448000, China.*

[2] *Zhuhai Beijing Institute of Technology (BIT), Beijing Institute of Technology, Zhuhai 519088, China.*

Supporting Information Contents:

Computational details.
Figure S1 Adsorption energy of CO_2 on M′M@NC catalysts.
Figure S2 Adsorption energy of CO on M′M@NC catalysts.
Figure S3 Adsorption energy of NO_2 on M′M@NC catalysts.
Figure S4 Adsorption energy of N_2 on M′M@NC catalysts.
Figure S5 Optimized geometrical structure of M′M@NC catalysts.
Figure S6 Optimized coupling configurations of *CO and *NH_x on M′M@NC catalysts.
Figure S7 Energy diagram of M′M@NC catalyst in ab initio molecular dynamics.
Figure S8 Free-energy diagram for OC-NH_x coupling process on M′M@NC catalysts.
Table S1 Hubbard correction of U for considered transitional metals.
Table S2 E_f and U_{diss} of M′M dimer embedded in N-doped graphene.
Table S3 E_{bind} and E_{agg} of M′M dimer embedded in N-doped graphene.
Table S4 Physical quantities chosen as M′M@NC features for SISSO program.
Table S5 Mathematical operators set in SISSO program.
Table S6 Mathematical descriptor of E_{coup} for C-N coupling process.
Table S7 Mathematical expression of RMSE and Pearson correlation matrix.
References

Computational details

Free energy calculations: free energy change is calculated as $\Delta E + \Delta ZPE - T\Delta S$. ΔE is the DFT calculated energy difference, and ΔZPE and $T\Delta S$ are the change of zero point energies and entropy calculated by the following equations:

$$ZPE = \sum_i \frac{1}{2} hv_i$$

$$S_{vib}(T) = R \sum_i \{ \frac{hv_i}{kT} \frac{e^{-\frac{hv_i}{kT}}}{1-e^{-\frac{hv_i}{kT}}} - \ln(1-e^{-\frac{hv_i}{kT}}) \}$$

The T is set to be 298.15K.

Kinetics computations: The transition states are obtained by using the climbing image nudged elastic band approach (CINEB). All transition states have been verified by vibrational frequency calculations with only one imaginary frequency. The values of energy barrier have been corrected by considering the zero-point energy with no configurational entropies included according to the transition state theory.

D-band center: To analyze the adsorption ability of catalysts quantitatively, the d-band center is calculated as follows

$$\varepsilon_d = \frac{\int_{-\infty}^{\infty} \varepsilon \rho_d d\varepsilon}{\int_{-\infty}^{\infty} \rho_d \, d\varepsilon}$$

Here, ε denotes the energy level, and ρ_d represents the projected density of d-states (PDOS) of the catalyst surface atoms. The d-band center, defined as the first moment of the d-state density relative to the Fermi level, serves as a widely accepted electronic descriptor for adsorption strength. In general, a higher d-band center (i.e., closer to the Fermi level) correlates with a stronger adsorption interaction between the catalyst surface and adsorbates, owing to the increased availability of antibonding states for hybridization.

Bond strength analysis: To elucidate the electronic origin of chemical bonding interactions, projected Crystal Orbital Hamilton Population (pCOHP) analyses were performed using the LOBSTER computational package[1-3]. The bonding and antibonding contributions between specific atom pairs were quantitatively evaluated through integration of the pCOHP curves, yielding integrated pCOHP (IpCOHP) values. These metrics provide a rigorous quantification of orbital-resolved bonding strength and enable detailed insight into the nature of metal–adsorbate interactions at the atomic scale.

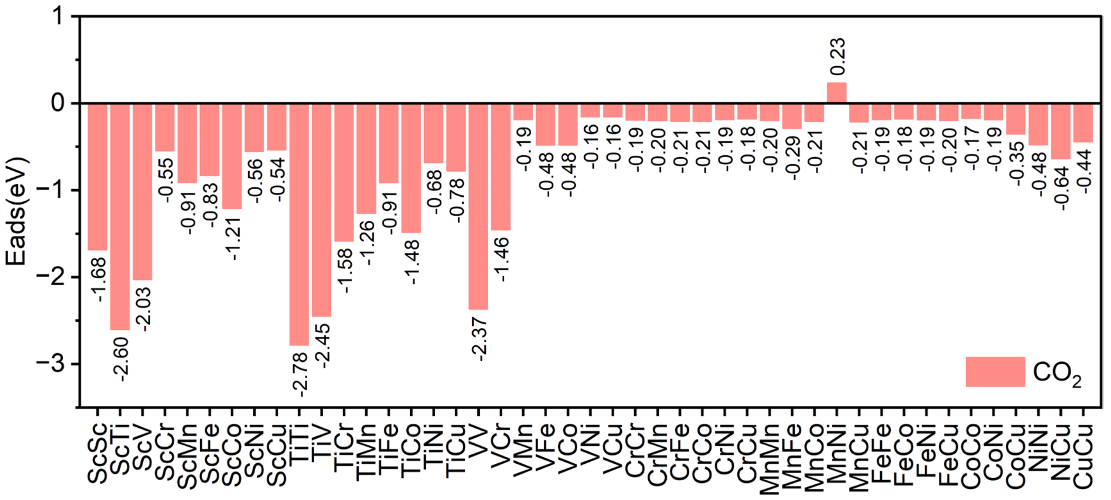

Figure S1. Adsorption energy of CO_2 on M′M@NC catalysts.

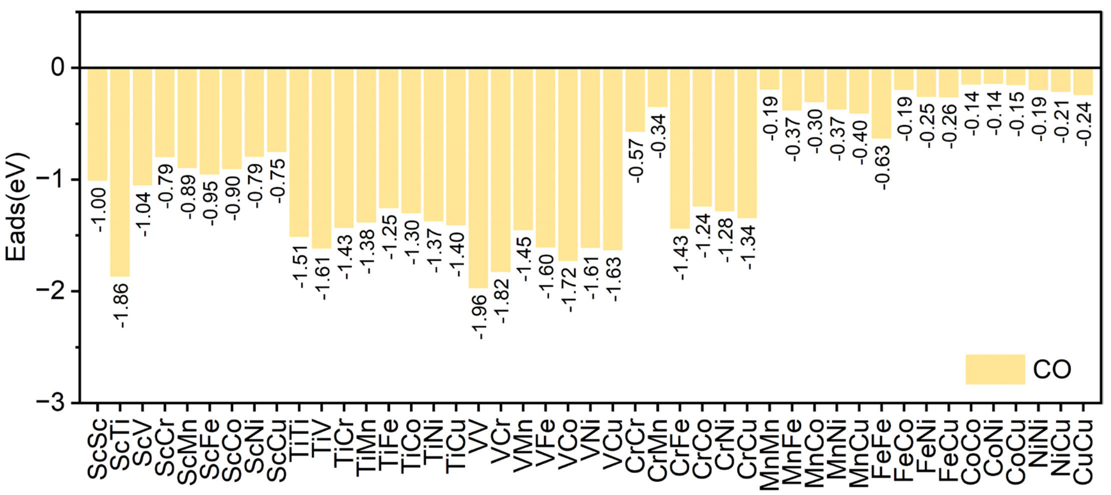

Figure S2. Adsorption energy of CO on M′M@NC catalysts.

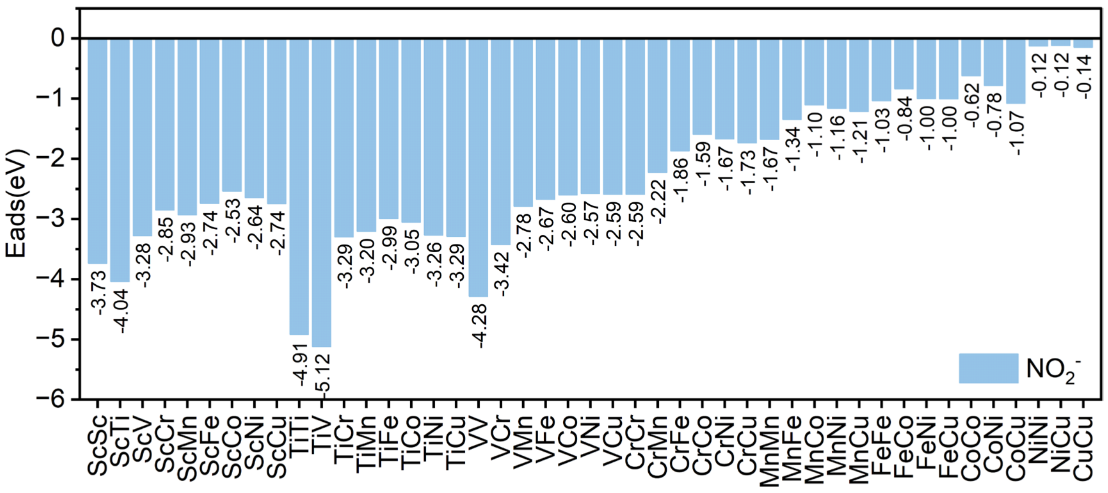

Figure S3. Adsorption energy of NO_2^- on M′M@NC catalysts.

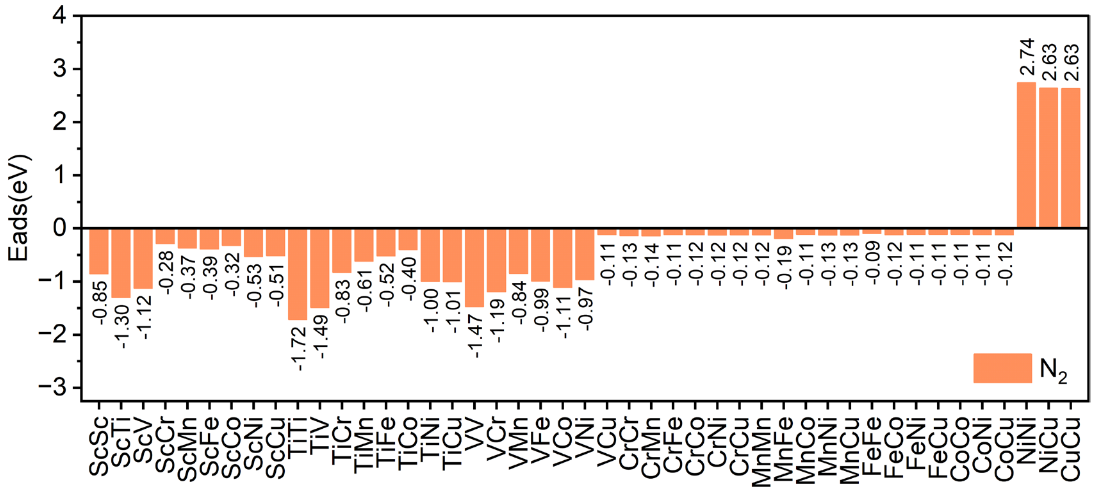

Figure S4. Adsorption energy of N_2 on M′M@NC catalysts.

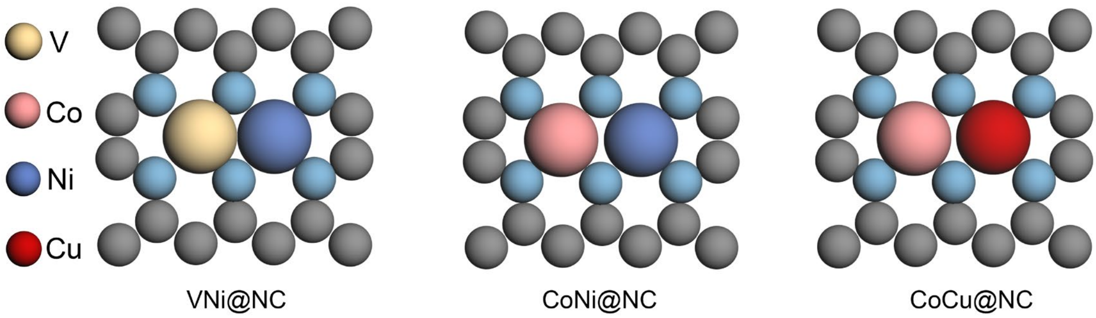

Figure S5. Optimized geometrical structure VNi@NC, CoNi@NC and CoCu@NC catalysts.

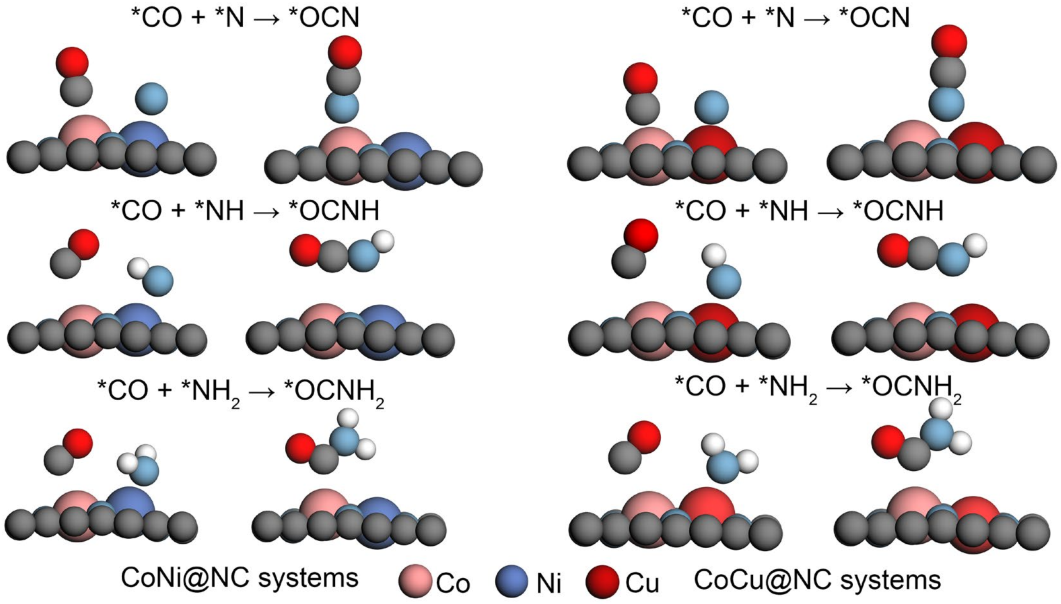

Figure S6. Optimized coupling configurations of *CO and *NH_x on CoNi@NC and CoCu@NC catalysts.

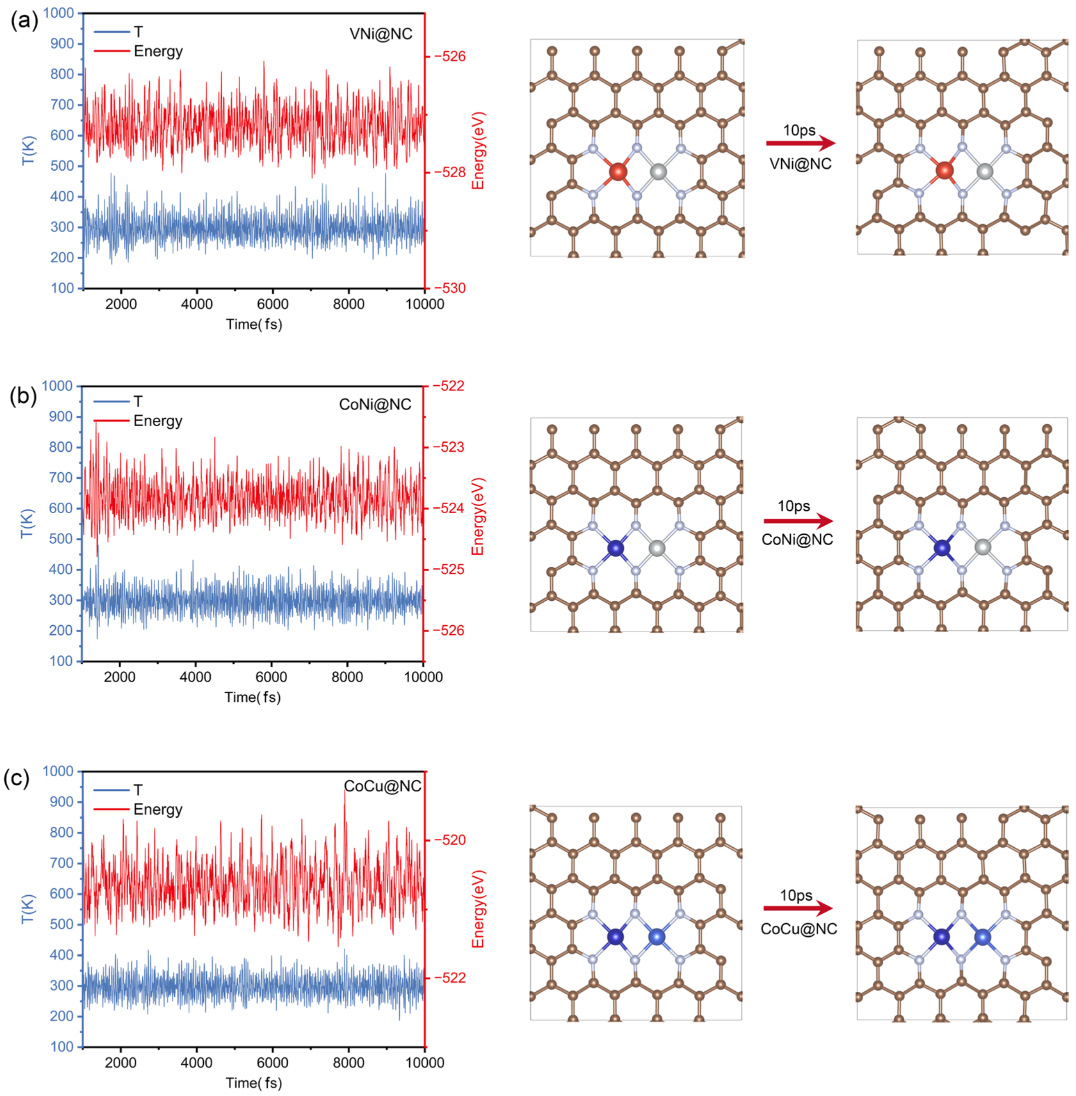

Figure S7. Variations of temperature and total energy as function of time for (a) VNi@NC, (b) CoNi@NC and (c) CoCu@NC in AIMD simulation within the NVT ensemble at 300K. The top views of their relaxed structures after 10 ps of simulation are given.

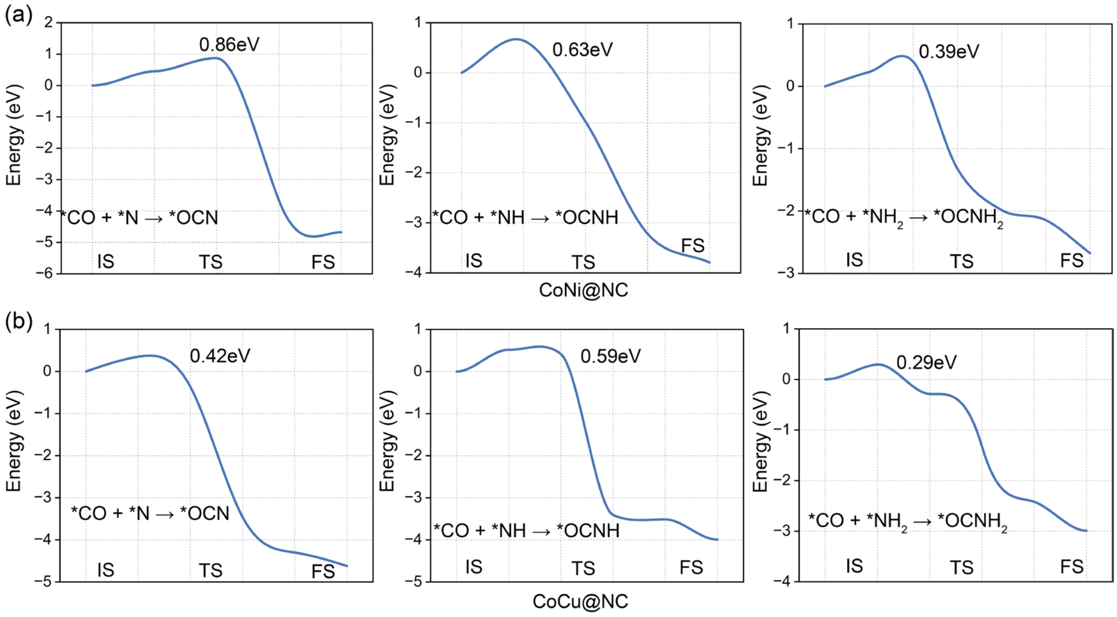

Figure S8. Free-energy diagram for OC-N, OC-NH and OC-NH_2 coupling process on (a) CoNi@NC and (b) CoCu@NC with climbing-image nudged elastic band approach (CI-NEB). Transition-state (TS) energy barriers are given.

Table S1. Hubbard correction of U for considered transitional metals.

M	U	ref
Sc	6.0	[4]
Ti	4.0	[4]
V	3.1	[5]
Cr	3.5	[5]
Mn	4.0	[5]
Fe	4.0	[5]
Co	3.2	[5]
Ni	6.4	[5]
Cu	4.0	[5]

Table S2. Computed formation energy (E_f) and dissolution potential (U_{diss}) of M′M dimer embedded in N-doped graphene.

M'M	E_f (eV)	U_{diss} (V)	M′M	E_f (eV)	U_{diss} (V)
ScSc	-5.43	0.09	VCu	-3.61	0.62
ScTi	-5.14	-0.02	CrCr	-4.23	1.20
ScV	-4.86	-0.13	CrMn	-4.42	1.02
ScCr	-4.84	-0.14	CrFe	-4.30	1.24
ScMn	-4.91	-0.11	CrCo	-4.34	1.26
ScFe	-4.68	-0.21	CrNi	-4.16	1.17
ScCo	-4.75	-0.18	CrCu	-3.41	0.80
ScNi	-4.85	-0.14	MnMn	-4.49	1.05
ScCu	-4.19	-0.40	MnFe	-4.33	0.97
TiTi	-4.86	0.80	MnCo	-4.31	0.96
TiV	-4.57	0.66	MnNi	-4.00	0.81
TiCr	-4.57	0.65	MnCu	-3.27	0.45
TiMn	-4.76	0.75	FeFe	-4.09	1.59
TiFe	-4.73	0.73	FeCo	-3.96	1.53
TiCo	-4.73	0.73	FeNi	-3.71	1.41
TiNi	-4.67	0.71	FeCu	-2.97	1.04
TiCu	-3.96	0.35	CoCo	-3.85	1.67
VV	-4.20	0.92	CoNi	-3.65	1.57
VCr	-4.21	0.93	CoCu	-2.82	1.16
VMn	-4.61	1.12	NiNi	-3.69	1.58
VFe	-4.39	1.02	NiCu	-2.97	1.22
VCo	-4.41	1.02	CuCu	-2.47	1.57
VNi	-4.33	0.99			

Table S3. Computed binding energy (E_{bind}) and aggregation energy (E_{agg}) of M′M dimer embedded in N-doped graphene.

M'M	E_{bind} (eV)	E_{agg} (eV)	M′M	E_{bind} (eV)	E_{agg} (eV)
ScSc	-10.85	-6.64	VCu	-7.21	-2.78
ScTi	-10.28	-5.53	CrCr	-8.45	-4.38
ScV	-9.73	-4.94	CrMn	-8.83	-4.88
ScCr	-9.68	-5.54	CrFe	-8.60	-4.13
ScMn	-9.83	-5.80	CrCo	-8.69	-4.19
ScFe	-9.37	-4.83	CrNi	-8.32	-3.88
ScCo	-9.50	-4.94	CrCu	-6.82	-3.03
ScNi	-9.70	-5.19	MnMn	-8.97	-5.13
ScCu	-8.38	-4.52	MnFe	-8.66	-4.31
TiTi	-9.72	-4.42	MnCo	-8.62	-4.24
TiV	-9.15	-3.82	MnNi	-8.01	-3.68
TiCr	-9.14	-4.45	MnCu	-6.54	-2.87
TiMn	-9.52	-4.95	FeFe	-8.17	-3.31
TiFe	-9.45	-4.37	FeCo	-7.92	-3.03
TiCo	-9.45	-4.34	FeNi	-7.43	-2.59
TiNi	-9.34	-4.29	FeCu	-5.95	-1.76
TiCu	-7.92	-3.51	CoCo	-7.70	-2.78
VV	-8.39	-3.03	CoNi	-7.29	-2.43
VCr	-8.43	-3.71	CoCu	-5.64	-1.42
VMn	-9.23	-4.63	NiNi	-7.37	-2.56
VFe	-8.79	-3.68	NiCu	-5.94	-1.78
VCo	-8.82	-3.68	CuCu	-4.93	-1.42
VNi	-8.66	-3.58			

Table S4. List of collected 48 physical quantities chosen as M′M@NC features for SISSO program.

Physical quantities	Averaging	Subtraction
Atomic Number	AM_A	AM_D
Atomic Weight	AW_A	AW_D
Group	G_A	G_D
Mendeleev Number	MN_A	MN_D
Atomic Radius	AR_A	AR_D
Covalent Radius	CR_A	CR_D
Pauling Electronegativity	EL_A	EL_D
Mulliken EN	MEN_A	MEN_D
Metallic valence	MV_A	MV_D
s valence electrons	N_{sA}	N_{sD}
p valence electrons	N_{pA}	N_{pD}
d valence electrons	N_{dA}	N_{dD}
1st ionization potential	$1IP_A$	$1IP_D$
Polarizability	P_A	P_D
Melting Point	T_{MA}	T_{MD}
Boiling Point	TB_A	TB_D
Density	D_A	D_D
Specific heat	SH_A	SH_D
Heat of vaporization	HV_A	HV_D
Thermal conductivity	TC_A	TC_D
Heat atomization	HA_A	HA_D
Cohesive energy	CE_A	CE_D
Magmom	M_A	M_D
SpaceGroup Number	SG_A	SG_D

Table S5. Mathematical operators set in SISSO program.

Mathematical operators	Description
+	Addition
-	Subtraction
*	Multiplication
/	Division
Exp()	Exponential function
Exp(-)	Negative Exponential function
^-1	Negative first power
^2	Square power
^3	Cube power
sqrt	Square root
cbrt	Cube root
log	Natural logarithm

Table S6. Mathematical descriptor of E_{coup} for all considered coupling process.

Process	Mathematical descriptor
OC-N	$(0.15\times10^{-2})(T_{MA}-2T_{MD})\frac{N_{dA}^{3}}{e^{N_{dA}}}+(0.34\times10^{3})\left(\frac{T_{MD}^{2}}{T_{MA}^{3}(EL_{D}^{2}-e^{-MN_{D}})}\right)-0.43$
OC-NH	$(0.13\times10^{-3})T_{MA}N_{dA}^{\frac{3}{2}}(MN_{D}EL_{D}-e^{EL_{D}}) + (-0.33\times10^{-2})(T_{MA}N_{dD}-T_{MD}N_{dA})\left(EL_{D}^{\frac{1}{3}}-e^{-MN_{D}}\right)+0.60$
OC-NH2	$(0.25\times10^{1})\frac{N_{dD}^{2}}{N_{dA}AW_{D}}\frac{lnT_{MD}}{lnN_{dA}}+(-0.52\times10^{0})\left(\frac{\frac{N_{dA}}{T_{MD}}e^{N_{dd}}}{\frac{N_{dA}}{N_{dD}}-\ln(MN_{D})}\right)-3.73$

Table S7. Expression of root mean square error (RMSE) and Pearson correlation matrix.

Function	Mathematical expression
root mean square error (RMSE)	$\sqrt{\frac{\sum_{i=1}^{n}(y_i - f(x_i))^2}{n}}$
Pearson correlation	$\frac{\sum_{i=1}^{n}(Y_i - \bar{Y})(X_i - \bar{X})}{\sqrt{\sum_{i=1}^{n}(Y_i - \bar{Y})^2}\sqrt{\sum_{i=1}^{n}(X_i - \bar{X})^2}}$

Where y_i, x_i, $f(x_i)$, n, represent the target data value, the feature, the predicted value and the sample numbers, respectively. And $\bar{X}$ and $\bar{Y}$ are the average value of two features while X_i and Y_i represent the predicted value of two features in the data set.

References

[1] V. L. Deringer, A. L. Tchougréeff and R. Dronskowski, *J. Phys. Chem. A* **2011**, 115, 5461.

[2] R. Dronskowski and P. E. Bloechl, *J. Phys. Chem.* **1993**, 97, 8617.

[3] R. Nelson, C. Ertural, J. George, V. L. Deringer, G. Hautier and R. Dronskowski, *J. Comput. Chem.* **2020**, 41, 1931–1940.

[4] M. Kanoun, S. Goumri-Said, U. Schwingenschlögl, A. Manchon, *Chem. Phys. Lett.* **2012**, 532, 96.

[5] L. Wang, *Phys. Rev. B*, **2006**, 73, 195107.
